# Bis(η^6^-naphthalene)­molybdenum(0)

**DOI:** 10.1107/S1600536812002504

**Published:** 2012-01-31

**Authors:** Mikhail E. Minyaev, John E. Ellis, William J. Wolf

**Affiliations:** a Institute of Organoelement Compounds, 28 Vavilova Str., 117813 Moscow, Russian Federation; b Department of Chemistry, University of Minnesota, Minneapolis, 207 Pleasant Str. SE, MN 55455, USA

## Abstract

The title compound, [Mo(C_10_H_8_)_2_], was prepared from the naphthalene radical anion and MoCl_4_(thf)_2_ (thf is tetra­hydro­furan). In the crystal, the mol­ecule is located on an inversion center. The Mo atom is equally disordered over two positions; the range of Mo—C distances is 2.2244 (19)–2.3400 (17) Å for both components of the disorder.

## Related literature

For background to transition metal–arene complexes, see: Seyferth (2002*a* ▶,*b* ▶). For the structures of the isotypic Cr and V complexes, see: Elschenbroich *et al.* (1982 ▶); Pomije *et al.* (1997 ▶). For the structures of homoleptic naphthalenate ate-complexes, see: Jang & Ellis (1994 ▶); Brennessel *et al.* (2002 ▶, 2006 ▶). For the preparation of the title compound, see: Kündig & Timms (1977 ▶); Thi *et al.* (1992 ▶); Pomije *et al.* (1997 ▶).
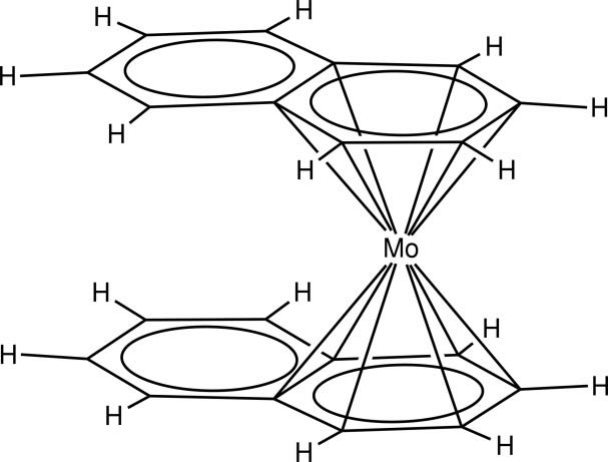


## Experimental

### 

#### Crystal data

[Mo(C_10_H_8_)_2_]
*M* *_r_* = 352.27
Monoclinic, 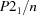

*a* = 8.4452 (10) Å
*b* = 8.0716 (10) Å
*c* = 10.9890 (13) Å
β = 109.186 (2)°
*V* = 707.47 (15) Å^3^
*Z* = 2
Mo *K*α radiation
μ = 0.92 mm^−1^
*T* = 173 K
0.60 × 0.60 × 0.30 mm

#### Data collection

Bruker SMART Platform CCD diffractometer
Absorption correction: multi-scan (*SADABS*; Bruker, 2003 ▶) *T*_min_ = 0.610, *T*_max_ = 0.771
8208 measured reflections
1674 independent reflections
1420 reflections with *I* > 2σ(*I*)
*R*_int_ = 0.031

#### Refinement

*R*[*F* ^2^ > 2σ(*F* ^2^)] = 0.028
*wR*(*F* ^2^) = 0.069
*S* = 1.03
1674 reflections
132 parameters
All H-atom parameters refined
Δρ_max_ = 0.27 e Å^−3^
Δρ_min_ = −0.28 e Å^−3^

### 

Data collection: *SMART* (Bruker, 2003 ▶); cell refinement: *SAINT* (Bruker, 2003 ▶); data reduction: *SAINT*; program(s) used to solve structure: *SHELXS97* (Sheldrick, 2008 ▶); program(s) used to refine structure: *SHELXL97* (Sheldrick, 2008 ▶); molecular graphics: *SHELXTL* (Sheldrick, 2008 ▶); software used to prepare material for publication: *SHELXTL*.

## Supplementary Material

Crystal structure: contains datablock(s) I, global. DOI: 10.1107/S1600536812002504/tk5041sup1.cif

Structure factors: contains datablock(s) I. DOI: 10.1107/S1600536812002504/tk5041Isup2.hkl

Supplementary material file. DOI: 10.1107/S1600536812002504/tk5041Isup3.mol

Additional supplementary materials:  crystallographic information; 3D view; checkCIF report

## Figures and Tables

**Fig. 1.**
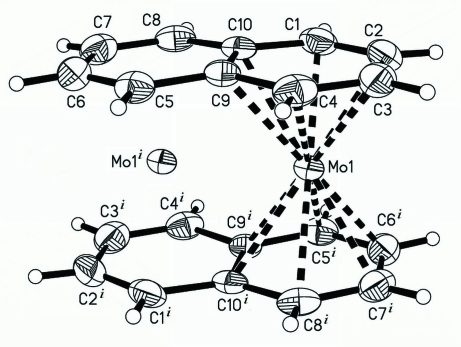
Molecular structure of Mo(C10H8)2 (50% atomic displacement parameters). The asymmetric unit consists of one half of the molecule. The occupancies of Mo1 and Mo1i sites are 50%. Symmetry code: (i) -x + 1, -y, -z + 2.

## supplementary crystallographic information

### Comment

Studies of classical arene complexes of transition elements have had significant impact on understanding metal – organic ligand bonding (Seyferth, 2002*a*,*b*). However, only a few molecular structures of d-metal neutral homoleptic naphthalene species, i.e. Cr(η^6^-C_10_H_8_)_2_ (Elschenbroich *et al.*, 1982), V(η^6^-C_10_H_8_)_2_ (Pomije *et al.*, 1997) and homoleptic naphthalene *ate*-complexes [Zr(η^4^-C_10_H_8_)_3_]^2-^ (Jang *et al.*, 1994), [Ta(η^4^-C_10_H_8_)_3_]^-^ (Brennessel *et al.*, 2002) and [Co(η^4^-C_10_H_8_)_2_]^-^ (Brennessel *et al.*, 2006), have been established.

Previous examples of neutral homoleptic naphthalene complexes of transition metals were obtained by metal-atom-ligand-vapor co-condensation synthesis (Kündig *et al.*, 1977). The title compound, Mo(η^6^-C_10_H_8_)_2_, was later obtained by reduction of MoCl_5_ or MoCl_4_(thf)_2_ with Mg, followed by evaporation of Mo atoms along with free naphthalene under vacuum (Thi *et al.*, 1992), or by reduction of MoCl_4_(thf)_2_ or MoCl_3_(thf)_3_ with lithium naphthalenide (Pomije *et al.*, 1997).

We prepared Mo(C_10_H_8_)_2_ from LiC_10_H_8_ and MoCl_4_(thf)_2_ under argon in THF media in 13% yield. The ^1^H NMR spectrum is identical with previously described by Pomije *et al.* (1997). The Mo(C_10_H_8_)_2_ crystal structure is isomorphous with previously published structures of Cr(C_10_H_8_)_2_ (Elschenbroich *et al.*, 1982) and V(C_10_H_8_)_2_ (Pomije *et al.*, 1997). The molecule is located on the inversion center, one half of the molecule is unique. The molybdenum atom is disordered equally between two positions with occupancy of 50%. The C–C distances are similar to those found in Cr(C_10_H_8_)_2_ and V(C_10_H_8_)_2_. The Mo–centroid(C_6_) distances are 1.782 Å (C1—C2—C3—C4—C9—C10) and 1.770 Å (C5—C6—C7—C8—C10—C9) and centroid(C_6_)–Mo–centroid(C_6_) angle is 178°. The naphthalene ligand is slightly bent along the C9—C10 bond towards molybdenum atom, the angle between flat rings in the naphthalene ligand is 177°.

### Experimental

All synthetic manipulations were carried out in atmosphere of purified argon or under vacuum, using Schlenk type glassware and dry box techniques. Freshly cut lithium wire (0.34 g, 49 mmol), sublimed naphthalene (9.15 g, 71.4 mmol) and THF (200 ml) were added to a 1 *L* round-bottomed flask equipped with a glass covered stirbar. The mixture was stirred for 17 h at ambient temperature, then the resulting deep green solution was cooled to -60°C. A cold (-60°C) suspension of MoCl_4_(thf)_2_ (4.5 g, 11.8 mmol) and C_10_H_8_ (6.10 g, 47.6 mmol) in THF (100 ml) was added dropwise to the cold solution of lithium naphthalenide *via* large cannula. The reaction mixture was allowed to warm to ambient temperature over 15 h. It was filtered to provide a dark red solution, then THF was removed under vacuum. The resulting solid was stirred in toluene (200 ml), the mixture was filtered, and all toluene was removed from the solution under vacuum. The remaining solid was transferred to a sublimator, and an excess of naphthalene was removed by sublimation under vacuum at 30°C over three days. The remaining purple brown solid was taken up in toluene (100 ml) and the solution was filtered. All but 5 ml of toluene was removed under vacuum, pentane (100 ml) was added. The resulting mixture was cooled to -78°C and stirred. The precipitate was filtered off to give Mo(C_10_H_8_)_2_ (0.539 g, 1.53 mmol, 13.0%) as a dark purple microcrystalline solid. ^1^H NMR (300 MHz, C_6_D_6_, 25°C): δ (p.p.m.) = 4.67 (m, 2H). 5.02 (m, 2H), 6.56 (m, 2H), 6.78 (m, 2H) p.p.m..

X-ray quality single crystals were grown at 0°C over a 2 week period by slow diffusion of pentane (100 ml) into a nearly saturated solution of Mo(C_10_H_8_)_2_ (100 mg) in toluene (c.a. 20 ml).

### Refinement

The C—H atoms were refined freely; range of C—H distances = 0.89 (2) to 0.94 (2) Å. The Mo atom is disordered equally between two positions with occupancy of 50% each.

### Crystal data

**Table:**

[Mo(C_10_H_8_)_2_]	*F*(000) = 356
*M**_r_* = 352.27	*D*_x_ = 1.654 Mg m^−^^3^
Monoclinic, *P*2_1_/*n*	Mo *K*α radiation, λ = 0.71073 Å
Hall symbol: -P 2yn	Cell parameters from 2191 reflections
*a* = 8.4452 (10) Å	θ = 2.7–28.3°
*b* = 8.0716 (10) Å	µ = 0.92 mm^−^^1^
*c* = 10.9890 (13) Å	*T* = 173 K
β = 109.186 (2)°	Block, dark-red
*V* = 707.47 (15) Å^3^	0.60 × 0.60 × 0.30 mm
*Z* = 2	

### Data collection

**Table:**

Bruker SMART Platform CCD diffractometer	1674 independent reflections
Radiation source: normal-focus sealed tube	1420 reflections with *I* > 2σ(*I*)
graphite	*R*_int_ = 0.031
area detector, ω and φ scans	θ_max_ = 27.9°, θ_min_ = 2.7°
Absorption correction: multi-scan (*SADABS*; Bruker, 2003)	*h* = −10→11
*T*_min_ = 0.610, *T*_max_ = 0.771	*k* = −10→10
8208 measured reflections	*l* = −14→14

### Refinement

**Table:**

Refinement on *F*^2^	Primary atom site location: structure-invariant direct methods
Least-squares matrix: full	Secondary atom site location: difference Fourier map
*R*[*F*^2^ > 2σ(*F*^2^)] = 0.028	Hydrogen site location: inferred from neighbouring sites
*wR*(*F*^2^) = 0.069	All H-atom parameters refined
*S* = 1.03	*w* = 1/[σ^2^(*F*_o_^2^) + (0.0357*P*)^2^ + 0.251*P*] where *P* = (*F*_o_^2^ + 2*F*_c_^2^)/3
1674 reflections	(Δ/σ)_max_ = 0.001
132 parameters	Δρ_max_ = 0.27 e Å^−^^3^
0 restraints	Δρ_min_ = −0.28 e Å^−^^3^

### Special details

**Table:**

Geometry. All e.s.d.'s (except the e.s.d. in the dihedral angle between two l.s. planes) are estimated using the full covariance matrix. The cell e.s.d.'s are taken into account individually in the estimation of e.s.d.'s in distances, angles and torsion angles; correlations between e.s.d.'s in cell parameters are only used when they are defined by crystal symmetry. An approximate (isotropic) treatment of cell e.s.d.'s is used for estimating e.s.d.'s involving l.s. planes.
Refinement. Refinement of *F*^2^ against ALL reflections. The weighted *R*-factor *wR* and goodness of fit *S* are based on *F*^2^, conventional *R*-factors *R* are based on *F*, with *F* set to zero for negative *F*^2^. The threshold expression of *F*^2^ > σ(*F*^2^) is used only for calculating *R*-factors(gt) *etc*. and is not relevant to the choice of reflections for refinement. *R*-factors based on *F*^2^ are statistically about twice as large as those based on *F*, and *R*- factors based on ALL data will be even larger.

### Fractional atomic coordinates and isotropic or equivalent isotropic displacement parameters (Å^2^)

**Table:**

	*x*	*y*	*z*	*U*_iso_*/*U*_eq_	Occ. (<1)
Mo1	0.63424 (3)	0.01329 (3)	0.97463 (3)	0.02253 (11)	0.50
C1	0.4807 (3)	0.0582 (3)	0.76370 (18)	0.0382 (4)	
C2	0.6382 (3)	0.1252 (3)	0.7895 (2)	0.0481 (6)	
C3	0.7005 (3)	0.2429 (3)	0.8901 (2)	0.0452 (5)	
C4	0.6048 (2)	0.2901 (2)	0.96470 (19)	0.0358 (4)	
C9	0.4393 (2)	0.22530 (19)	0.93978 (16)	0.0261 (4)	
C5	0.3393 (2)	0.2642 (2)	1.01832 (17)	0.0318 (4)	
C6	0.1831 (3)	0.1938 (2)	0.99537 (19)	0.0358 (4)	
C7	0.1202 (2)	0.0780 (3)	0.89365 (19)	0.0372 (4)	
C8	0.2145 (2)	0.0343 (2)	0.81728 (17)	0.0330 (4)	
C10	0.3753 (2)	0.1064 (2)	0.83615 (15)	0.0277 (4)	
H1	0.442 (3)	−0.021 (2)	0.702 (2)	0.040 (6)*	
H2	0.707 (3)	0.091 (3)	0.744 (2)	0.058 (7)*	
H3	0.806 (3)	0.289 (3)	0.910 (2)	0.058 (7)*	
H4	0.641 (3)	0.363 (3)	1.032 (2)	0.037 (6)*	
H5	0.380 (2)	0.337 (2)	1.086 (2)	0.034 (5)*	
H6	0.118 (3)	0.218 (3)	1.048 (2)	0.047 (6)*	
H7	0.021 (3)	0.024 (3)	0.881 (2)	0.049 (7)*	
H8	0.179 (3)	−0.044 (3)	0.758 (2)	0.034 (5)*	

### Atomic displacement parameters (Å^2^)

**Table:**

	*U*^11^	*U*^22^	*U*^33^	*U*^12^	*U*^13^	*U*^23^
Mo1	0.02548 (16)	0.02166 (16)	0.01930 (16)	0.00090 (11)	0.00579 (11)	0.00237 (10)
C1	0.0496 (12)	0.0428 (11)	0.0217 (9)	0.0128 (9)	0.0111 (8)	0.0065 (8)
C2	0.0527 (13)	0.0604 (14)	0.0398 (12)	0.0210 (11)	0.0268 (11)	0.0232 (10)
C3	0.0342 (11)	0.0442 (12)	0.0579 (14)	0.0024 (9)	0.0160 (10)	0.0263 (10)
C4	0.0372 (11)	0.0238 (9)	0.0396 (11)	−0.0031 (7)	0.0036 (9)	0.0086 (8)
C9	0.0314 (9)	0.0194 (8)	0.0246 (8)	0.0017 (6)	0.0054 (7)	0.0048 (6)
C5	0.0420 (11)	0.0229 (8)	0.0276 (9)	0.0049 (7)	0.0078 (8)	−0.0004 (7)
C6	0.0368 (10)	0.0364 (10)	0.0359 (10)	0.0079 (8)	0.0143 (9)	0.0045 (8)
C7	0.0286 (10)	0.0366 (10)	0.0410 (11)	−0.0017 (8)	0.0043 (8)	0.0086 (8)
C8	0.0390 (10)	0.0259 (9)	0.0245 (9)	−0.0003 (7)	−0.0025 (8)	0.0005 (7)
C10	0.0350 (9)	0.0248 (8)	0.0194 (8)	0.0050 (7)	0.0038 (7)	0.0056 (6)

### Geometric parameters (Å, °)

**Table:**

Mo1—C1	2.2826 (19)	C4—C9	1.432 (3)
Mo1—C2	2.236 (2)	C9—C5	1.427 (3)
Mo1—C3	2.225 (2)	C5—C6	1.381 (3)
Mo1—C4	2.2466 (19)	C6—C7	1.419 (3)
Mo1—C9	2.3176 (16)	C7—C8	1.379 (3)
Mo1—C10	2.3400 (17)	C8—C10	1.429 (3)
Mo1—C5^i^	2.2495 (18)	C9—C10	1.451 (2)
Mo1—C6^i^	2.2244 (19)	C1—H1	0.91 (2)
Mo1—C7^i^	2.2284 (19)	C2—H2	0.93 (2)
Mo1—C8^i^	2.2557 (18)	C3—H3	0.92 (3)
Mo1—C9^i^	2.3158 (16)	C4—H4	0.91 (2)
Mo1—C10^i^	2.3182 (16)	C5—H5	0.92 (2)
C1—C10	1.429 (3)	C6—H6	0.94 (2)
C1—C2	1.377 (3)	C7—H7	0.91 (3)
C2—C3	1.421 (3)	C8—H8	0.89 (2)
C3—C4	1.380 (3)		
			
C6^i^—Mo1—C3	115.50 (8)	C2—C1—Mo1	70.43 (12)
C6^i^—Mo1—C7^i^	37.18 (7)	C10—C1—Mo1	74.19 (10)
C3—Mo1—C7^i^	103.80 (8)	C1—C2—C3	120.6 (2)
C6^i^—Mo1—C2	102.80 (8)	C1—C2—Mo1	74.12 (12)
C3—Mo1—C2	37.15 (9)	C3—C2—Mo1	71.01 (12)
C7^i^—Mo1—C2	115.83 (8)	C4—C3—C2	120.5 (2)
C6^i^—Mo1—C4	144.57 (7)	C4—C3—Mo1	72.87 (11)
C3—Mo1—C4	35.95 (8)	C2—C3—Mo1	71.84 (12)
C7^i^—Mo1—C4	115.15 (7)	C3—C4—C9	120.73 (19)
C2—Mo1—C4	65.70 (8)	C3—C4—Mo1	71.18 (12)
C6^i^—Mo1—C5^i^	35.95 (7)	C9—C4—Mo1	74.44 (10)
C3—Mo1—C5^i^	143.88 (8)	C5—C9—C4	122.59 (17)
C7^i^—Mo1—C5^i^	65.64 (7)	C5—C9—C10	118.58 (16)
C2—Mo1—C5^i^	113.74 (8)	C4—C9—C10	118.68 (17)
C4—Mo1—C5^i^	179.14 (7)	C5—C9—Mo1^i^	69.28 (9)
C6^i^—Mo1—C8^i^	65.65 (7)	C4—C9—Mo1^i^	126.95 (11)
C3—Mo1—C8^i^	115.38 (8)	C10—C9—Mo1^i^	71.85 (9)
C7^i^—Mo1—C8^i^	35.82 (7)	C5—C9—Mo1	126.30 (11)
C2—Mo1—C8^i^	144.91 (9)	C4—C9—Mo1	69.04 (10)
C4—Mo1—C8^i^	103.61 (7)	C10—C9—Mo1	72.69 (9)
C5^i^—Mo1—C8^i^	77.22 (7)	Mo1^i^—C9—Mo1	65.77 (4)
C6^i^—Mo1—C1	113.87 (7)	C6—C5—C9	121.25 (17)
C3—Mo1—C1	65.22 (9)	C6—C5—Mo1^i^	71.04 (11)
C7^i^—Mo1—C1	144.00 (8)	C9—C5—Mo1^i^	74.33 (9)
C2—Mo1—C1	35.45 (8)	C5—C6—C7	120.16 (19)
C4—Mo1—C1	77.05 (7)	C5—C6—Mo1^i^	73.01 (11)
C5^i^—Mo1—C1	102.12 (7)	C7—C6—Mo1^i^	71.56 (11)
C8^i^—Mo1—C1	179.33 (7)	C8—C7—C6	120.40 (19)
C6^i^—Mo1—C9^i^	65.18 (7)	C8—C7—Mo1^i^	73.17 (11)
C3—Mo1—C9^i^	179.04 (7)	C6—C7—Mo1^i^	71.26 (11)
C7^i^—Mo1—C9^i^	77.15 (7)	C7—C8—C10	121.25 (17)
C2—Mo1—C9^i^	142.34 (8)	C7—C8—Mo1^i^	71.01 (11)
C4—Mo1—C9^i^	143.76 (7)	C10—C8—Mo1^i^	74.20 (10)
C5^i^—Mo1—C9^i^	36.39 (6)	C8—C10—C1	122.96 (17)
C8^i^—Mo1—C9^i^	65.46 (6)	C8—C10—C9	118.33 (16)
C1—Mo1—C9^i^	113.94 (7)	C1—C10—C9	118.56 (17)
C6^i^—Mo1—C9	178.65 (7)	C8—C10—Mo1^i^	69.43 (10)
C3—Mo1—C9	65.07 (7)	C1—C10—Mo1^i^	126.69 (12)
C7^i^—Mo1—C9	144.14 (7)	C9—C10—Mo1^i^	71.66 (9)
C2—Mo1—C9	76.91 (7)	C8—C10—Mo1	127.15 (11)
C4—Mo1—C9	36.52 (6)	C1—C10—Mo1	69.81 (10)
C5^i^—Mo1—C9	142.94 (7)	C9—C10—Mo1	71.02 (9)
C8^i^—Mo1—C9	115.35 (7)	Mo1^i^—C10—Mo1	65.37 (4)
C1—Mo1—C9	65.12 (7)	C2—C1—H1	120.8 (14)
C9^i^—Mo1—C9	114.23 (4)	C10—C1—H1	118.2 (14)
C6^i^—Mo1—C10^i^	77.33 (7)	Mo1—C1—H1	126.2 (13)
C3—Mo1—C10^i^	144.01 (8)	C1—C2—H2	120.5 (16)
C7^i^—Mo1—C10^i^	65.09 (7)	C3—C2—H2	118.9 (16)
C2—Mo1—C10^i^	178.65 (8)	Mo1—C2—H2	125.0 (15)
C4—Mo1—C10^i^	114.97 (7)	C4—C3—H3	117.5 (15)
C5^i^—Mo1—C10^i^	65.57 (6)	C2—C3—H3	122.1 (15)
C8^i^—Mo1—C10^i^	36.37 (7)	Mo1—C3—H3	126.5 (15)
C1—Mo1—C10^i^	143.26 (7)	C3—C4—H4	123.6 (13)
C9^i^—Mo1—C10^i^	36.49 (6)	C9—C4—H4	115.7 (13)
C9—Mo1—C10^i^	102.93 (6)	Mo1—C4—H4	126.8 (13)
C6^i^—Mo1—C10	142.39 (7)	C6—C5—H5	119.4 (12)
C3—Mo1—C10	76.73 (7)	C9—C5—H5	119.4 (12)
C7^i^—Mo1—C10	179.42 (7)	Mo1^i^—C5—H5	127.2 (12)
C2—Mo1—C10	64.45 (7)	C5—C6—H6	121.2 (14)
C4—Mo1—C10	65.41 (6)	C7—C6—H6	118.6 (14)
C5^i^—Mo1—C10	113.79 (6)	Mo1^i^—C6—H6	125.7 (14)
C8^i^—Mo1—C10	144.17 (7)	C8—C7—H7	118.3 (15)
C1—Mo1—C10	35.99 (6)	C6—C7—H7	121.1 (15)
C9^i^—Mo1—C10	102.32 (6)	Mo1^i^—C7—H7	123.6 (14)
C9—Mo1—C10	36.29 (6)	C7—C8—H8	120.6 (13)
C10^i^—Mo1—C10	114.63 (4)	C10—C8—H8	118.1 (13)
C2—C1—C10	121.0 (2)	Mo1^i^—C8—H8	124.5 (13)
			
C6^i^—Mo1—C1—C2	−78.24 (15)	C9^i^—Mo1—C9—C4	151.44 (12)
C3—Mo1—C1—C2	30.08 (13)	C10^i^—Mo1—C9—C4	114.69 (11)
C7^i^—Mo1—C1—C2	−49.1 (2)	C10—Mo1—C9—C4	−131.12 (15)
C4—Mo1—C1—C2	66.11 (14)	C3—Mo1—C9—C10	101.84 (12)
C5^i^—Mo1—C1—C2	−114.10 (14)	C7^i^—Mo1—C9—C10	−179.35 (11)
C9^i^—Mo1—C1—C2	−150.44 (13)	C2—Mo1—C9—C10	64.44 (11)
C9—Mo1—C1—C2	102.75 (15)	C4—Mo1—C9—C10	131.12 (15)
C10^i^—Mo1—C1—C2	−179.30 (12)	C5^i^—Mo1—C9—C10	−47.76 (15)
C10—Mo1—C1—C2	131.9 (2)	C8^i^—Mo1—C9—C10	−150.53 (10)
C6^i^—Mo1—C1—C10	149.85 (11)	C1—Mo1—C9—C10	28.93 (10)
C3—Mo1—C1—C10	−101.83 (13)	C9^i^—Mo1—C9—C10	−77.43 (9)
C7^i^—Mo1—C1—C10	179.02 (11)	C10^i^—Mo1—C9—C10	−114.18 (8)
C2—Mo1—C1—C10	−131.9 (2)	C3—Mo1—C9—Mo1^i^	179.28 (8)
C4—Mo1—C1—C10	−65.80 (12)	C7^i^—Mo1—C9—Mo1^i^	−101.91 (11)
C5^i^—Mo1—C1—C10	113.99 (12)	C2—Mo1—C9—Mo1^i^	141.87 (8)
C9^i^—Mo1—C1—C10	77.65 (12)	C4—Mo1—C9—Mo1^i^	−151.44 (12)
C9—Mo1—C1—C10	−29.17 (10)	C5^i^—Mo1—C9—Mo1^i^	29.67 (11)
C10^i^—Mo1—C1—C10	48.78 (19)	C8^i^—Mo1—C9—Mo1^i^	−73.10 (7)
C10—C1—C2—C3	0.7 (3)	C1—Mo1—C9—Mo1^i^	106.36 (7)
Mo1—C1—C2—C3	−55.86 (17)	C9^i^—Mo1—C9—Mo1^i^	0.0
C10—C1—C2—Mo1	56.59 (17)	C10^i^—Mo1—C9—Mo1^i^	−36.75 (6)
C6^i^—Mo1—C2—C1	113.35 (13)	C10—Mo1—C9—Mo1^i^	77.43 (9)
C3—Mo1—C2—C1	−131.1 (2)	C4—C9—C5—C6	−177.00 (16)
C7^i^—Mo1—C2—C1	150.43 (12)	C10—C9—C5—C6	−1.4 (2)
C4—Mo1—C2—C1	−102.13 (14)	Mo1^i^—C9—C5—C6	−55.60 (15)
C5^i^—Mo1—C2—C1	77.16 (14)	Mo1—C9—C5—C6	−90.3 (2)
C8^i^—Mo1—C2—C1	179.02 (12)	C4—C9—C5—Mo1^i^	−121.39 (15)
C9^i^—Mo1—C2—C1	47.56 (19)	C10—C9—C5—Mo1^i^	54.17 (13)
C9—Mo1—C2—C1	−65.29 (13)	Mo1—C9—C5—Mo1^i^	−34.68 (12)
C10—Mo1—C2—C1	−29.00 (12)	C9—C5—C6—C7	1.2 (3)
C6^i^—Mo1—C2—C3	−115.54 (13)	Mo1^i^—C5—C6—C7	−55.95 (16)
C7^i^—Mo1—C2—C3	−78.46 (14)	C9—C5—C6—Mo1^i^	57.15 (15)
C4—Mo1—C2—C3	28.97 (12)	C5—C6—C7—C8	0.3 (3)
C5^i^—Mo1—C2—C3	−151.74 (12)	Mo1^i^—C6—C7—C8	−56.39 (16)
C8^i^—Mo1—C2—C3	−49.88 (19)	C5—C6—C7—Mo1^i^	56.64 (16)
C1—Mo1—C2—C3	131.10 (19)	C6—C7—C8—C10	−1.4 (3)
C9^i^—Mo1—C2—C3	178.66 (11)	Mo1^i^—C7—C8—C10	−56.93 (15)
C9—Mo1—C2—C3	65.81 (13)	C6—C7—C8—Mo1^i^	55.48 (16)
C10—Mo1—C2—C3	102.10 (13)	C7—C8—C10—C1	176.59 (16)
C1—C2—C3—C4	0.9 (3)	Mo1^i^—C8—C10—C1	121.15 (16)
Mo1—C2—C3—C4	−56.46 (16)	C7—C8—C10—C9	1.2 (2)
C1—C2—C3—Mo1	57.35 (17)	Mo1^i^—C8—C10—C9	−54.27 (13)
C6^i^—Mo1—C3—C4	−151.64 (12)	C7—C8—C10—Mo1^i^	55.44 (16)
C7^i^—Mo1—C3—C4	−114.00 (13)	C7—C8—C10—Mo1	88.0 (2)
C2—Mo1—C3—C4	131.25 (19)	Mo1^i^—C8—C10—Mo1	32.57 (12)
C5^i^—Mo1—C3—C4	178.57 (12)	C2—C1—C10—C8	−176.75 (16)
C8^i^—Mo1—C3—C4	−77.86 (14)	Mo1—C1—C10—C8	−121.91 (16)
C1—Mo1—C3—C4	102.48 (14)	C2—C1—C10—C9	−1.3 (3)
C9—Mo1—C3—C4	29.72 (11)	Mo1—C1—C10—C9	53.51 (13)
C10^i^—Mo1—C3—C4	−47.57 (18)	C2—C1—C10—Mo1^i^	−89.0 (2)
C10—Mo1—C3—C4	66.25 (12)	Mo1—C1—C10—Mo1^i^	−34.14 (14)
C6^i^—Mo1—C3—C2	77.11 (14)	C2—C1—C10—Mo1	−54.85 (17)
C7^i^—Mo1—C3—C2	114.75 (13)	C5—C9—C10—C8	0.3 (2)
C4—Mo1—C3—C2	−131.25 (19)	C4—C9—C10—C8	176.00 (15)
C5^i^—Mo1—C3—C2	47.32 (19)	Mo1^i^—C9—C10—C8	53.20 (13)
C8^i^—Mo1—C3—C2	150.89 (12)	Mo1—C9—C10—C8	122.69 (14)
C1—Mo1—C3—C2	−28.78 (12)	C5—C9—C10—C1	−175.37 (15)
C9—Mo1—C3—C2	−101.53 (14)	C4—C9—C10—C1	0.4 (2)
C10^i^—Mo1—C3—C2	−178.82 (12)	Mo1^i^—C9—C10—C1	−122.43 (15)
C10—Mo1—C3—C2	−65.01 (13)	Mo1—C9—C10—C1	−52.93 (14)
C2—C3—C4—C9	−1.9 (3)	C5—C9—C10—Mo1^i^	−52.94 (13)
Mo1—C3—C4—C9	−57.84 (15)	C4—C9—C10—Mo1^i^	122.80 (14)
C2—C3—C4—Mo1	55.97 (17)	Mo1—C9—C10—Mo1^i^	69.50 (4)
C6^i^—Mo1—C4—C3	47.69 (19)	C5—C9—C10—Mo1	−122.43 (14)
C7^i^—Mo1—C4—C3	78.55 (14)	C4—C9—C10—Mo1	53.30 (13)
C2—Mo1—C4—C3	−29.87 (13)	Mo1^i^—C9—C10—Mo1	−69.50 (4)
C8^i^—Mo1—C4—C3	114.66 (13)	C6^i^—Mo1—C10—C8	67.8 (2)
C1—Mo1—C4—C3	−65.46 (13)	C3—Mo1—C10—C8	−177.41 (17)
C9^i^—Mo1—C4—C3	−178.45 (12)	C2—Mo1—C10—C8	145.24 (18)
C9—Mo1—C4—C3	−130.94 (18)	C4—Mo1—C10—C8	−141.19 (18)
C10^i^—Mo1—C4—C3	151.41 (12)	C5^i^—Mo1—C10—C8	39.17 (17)
C10—Mo1—C4—C3	−101.58 (14)	C8^i^—Mo1—C10—C8	−62.2 (2)
C6^i^—Mo1—C4—C9	178.64 (11)	C1—Mo1—C10—C8	116.7 (2)
C3—Mo1—C4—C9	130.94 (18)	C9^i^—Mo1—C10—C8	2.70 (17)
C7^i^—Mo1—C4—C9	−150.51 (11)	C9—Mo1—C10—C8	−111.65 (19)
C2—Mo1—C4—C9	101.07 (12)	C10^i^—Mo1—C10—C8	−33.67 (14)
C8^i^—Mo1—C4—C9	−114.40 (11)	C6^i^—Mo1—C10—C1	−48.81 (17)
C1—Mo1—C4—C9	65.49 (11)	C3—Mo1—C10—C1	65.93 (13)
C9^i^—Mo1—C4—C9	−47.50 (18)	C2—Mo1—C10—C1	28.59 (13)
C10^i^—Mo1—C4—C9	−77.64 (12)	C4—Mo1—C10—C1	102.16 (13)
C10—Mo1—C4—C9	29.36 (10)	C5^i^—Mo1—C10—C1	−77.49 (13)
C3—C4—C9—C5	176.78 (16)	C8^i^—Mo1—C10—C1	−178.89 (12)
Mo1—C4—C9—C5	120.50 (15)	C9^i^—Mo1—C10—C1	−113.95 (12)
C3—C4—C9—C10	1.2 (2)	C9—Mo1—C10—C1	131.69 (16)
Mo1—C4—C9—C10	−55.06 (13)	C10^i^—Mo1—C10—C1	−150.33 (13)
C3—C4—C9—Mo1^i^	89.3 (2)	C6^i^—Mo1—C10—C9	179.49 (11)
Mo1—C4—C9—Mo1^i^	33.05 (13)	C3—Mo1—C10—C9	−65.76 (11)
C3—C4—C9—Mo1	56.28 (16)	C2—Mo1—C10—C9	−103.11 (12)
C3—Mo1—C9—C5	−145.02 (17)	C4—Mo1—C10—C9	−29.54 (10)
C7^i^—Mo1—C9—C5	−66.2 (2)	C5^i^—Mo1—C10—C9	150.82 (10)
C2—Mo1—C9—C5	177.57 (17)	C8^i^—Mo1—C10—C9	49.42 (15)
C4—Mo1—C9—C5	−115.7 (2)	C1—Mo1—C10—C9	−131.69 (16)
C5^i^—Mo1—C9—C5	65.4 (2)	C9^i^—Mo1—C10—C9	114.35 (8)
C8^i^—Mo1—C9—C5	−37.40 (17)	C10^i^—Mo1—C10—C9	77.98 (9)
C1—Mo1—C9—C5	142.06 (17)	C6^i^—Mo1—C10—Mo1^i^	101.52 (11)
C9^i^—Mo1—C9—C5	35.70 (13)	C3—Mo1—C10—Mo1^i^	−143.74 (8)
C10^i^—Mo1—C9—C5	−1.05 (16)	C2—Mo1—C10—Mo1^i^	178.91 (8)
C10—Mo1—C9—C5	113.13 (19)	C4—Mo1—C10—Mo1^i^	−107.52 (7)
C3—Mo1—C9—C4	−29.28 (12)	C5^i^—Mo1—C10—Mo1^i^	72.84 (7)
C7^i^—Mo1—C9—C4	49.53 (16)	C8^i^—Mo1—C10—Mo1^i^	−28.56 (11)
C2—Mo1—C9—C4	−66.68 (12)	C1—Mo1—C10—Mo1^i^	150.33 (13)
C5^i^—Mo1—C9—C4	−178.88 (12)	C9^i^—Mo1—C10—Mo1^i^	36.37 (6)
C8^i^—Mo1—C9—C4	78.35 (12)	C9—Mo1—C10—Mo1^i^	−77.98 (9)
C1—Mo1—C9—C4	−102.19 (13)	C10^i^—Mo1—C10—Mo1^i^	0.0

Symmetry codes: (i) −*x*+1, −*y*, −*z*+2.

## References

[bb1] Brennessel, W. W., Ellis, J. E., Pomije, M. K., Sussman, V. J., Urnezius, E. & Young, V. G. (2002). *J. Am. Chem. Soc.* **124**, 10258–10259. 10.1021/ja020725y 12197710

[bb2] Brennessel, W. W., Young, V. G. & Ellis, J. E. (2006). *Angew. Chem. Int. Ed.* **45**, 7268–7271. 10.1002/anie.200602937 17024697

[bb3] Bruker (2003). *SADABS*, *SMART* and *SAINT* Bruker AXS Inc., Madison, Wisconsin, USA.

[bb4] Elschenbroich, C., Mockel, R., Massa, W., Birkhahn, M. & Zenneck, U. (1982). *Chem. Ber.* **115**, 334–345.

[bb5] Jang, M. & Ellis, J. E. (1994). *Angew. Chem. Int. Ed. Engl.* **33**, 1973–1975.

[bb6] Kündig, E. P. & Timms, P. L. (1977). *J. Chem. Soc. Chem. Commun.* pp. 912–913.

[bb7] Pomije, M. K., Kurth, C. J., Ellis, J. E. & Barybin, M. V. (1997). *Organometallics*, **16**, 3582–3587.

[bb8] Seyferth, D. (2002*a*). *Organometallics*, **21**, 1520–1530.

[bb9] Seyferth, D. (2002*b*). *Organometallics*, **21**, 2800–2820.

[bb10] Sheldrick, G. M. (2008). *Acta Cryst.* A**64**, 112–122. 10.1107/S0108767307043930 18156677

[bb11] Thi, N. P. D., Spichiger, S., Paglia, P., Bernardinelli, G., Kündig, E. P. & Timms, P. L. (1992). *Helv. Chim. Acta*, **75**, 2593–2607.

